# Physiological and Pathological Role of ROS: Benefits and Limitations of Antioxidant Treatment

**DOI:** 10.3390/ijms20194810

**Published:** 2019-09-27

**Authors:** Sergio Di Meo, Gaetana Napolitano, Paola Venditti

**Affiliations:** 1 Dipartimento di Biologia, Università di Napoli Federico II, Complesso Universitario Monte Sant’Angelo, Via Cinthia, I-80126 Napoli, Italy; 2 Dipartimento di Scienze e Tecnologie, Università degli Studi di Napoli Parthenope, via Acton n. 38-I-80133 Napoli, Italy; gaetana.napolitano@uniparthenope.it

From their discovery in biological systems, reactive oxygen species (ROS) have been considered key players in tissue injury for their capacity to oxidize biological macromolecules. Aerobic organisms possess a system of biochemical defenses to neutralize the oxidative effects of ROS, but the balance between ROS generation and the antioxidant system is slightly in favor of the ROS so that a continuous low level of oxidative damage exists [1]. When the imbalance toward the ROS increases, as happens under several conditions, oxidative stress arises. This has been related to the onset of many pathological conditions including cardiovascular disease, diabetes, rheumatoid arthritis, cancer, and neurodegenerative disorders [2]. It has been proposed that if ROS are involved in many pathological conditions, the use of exogenous antioxidants can help their management. However, starting from the end of the 1970s, increasing experimental evidence has led to an opposing view about the ROS’ role in biological systems. This suggests that living systems not only adapted to the coexistence with free radicals but developed methods to use them in critical physiological processes [2]. It has also been shown that, when the generation of ROS induces adaptive responses that are beneficial to the organism, the use of antioxidants can be detrimental [2].

The papers reported in this Special Issue deal with different aspects of reactive oxygen species (ROS) actions in living organisms. 

Some papers consider the role of ROS in inducing cellular dysfunction.

Thus, Hua et al. [3] treated human umbilical vein endothelial cells (HUVECs) with H_2_O_2_ to obtain a cell model of oxidative stress to study the role of sphingomyelin synthase 2 (SMS2) in endothelial disease (ED). They found that SMS2 induces the stress of the endoplasmic reticulum (ER) that leads to ED both activating the Wnt/β-catenin pathway and promoting intracellular cholesterol accumulation, both of which contribute to the induction of ER stress and finally lead to ED.

Querio et al. [4] used adult rat cardiomyocytes stressed with H_2_O_2_ or doxorubicin to verify if trimethylamine *N*-oxide (TMAO), an organic compound derived from dietary choline and L-carnitine, is a factor involved in the progression of atherosclerosis and other cardiovascular diseases. They show that TMAO does not affect the treatment’s effect on cell viability, sarcomere length, intracellular ROS, and mitochondrial membrane potential. Therefore, they conclude that TMAO cannot be considered a direct cause or an exacerbating risk factor of cardiac damage at the cellular level in acute conditions.

Another work evaluates the role of ROS as agents able to induce cellular protection. 

Lin et al. [5] demonstrated that ROS are involved in the mechanism underlying the protective action of carbon monoxide-releasing molecule 2 (CORM-2) against lipopolysaccharide (LPS)-induced inflammation in mice lung. CORM-2 induces the expression of heme oxygenase 1 (HO-1), a member of the heme oxygenase (HO) family, able to directly protect various organs from oxidative damages. This is due to the activation of protein kinase C (PKC)α and proline-rich tyrosine kinase (Pyk2), which, in turn, activate NOX-derived ROS generation. The ROS signal activates the extracellular signal-regulated kinase 1/2 (ERK1/2) that upregulates c-Fos and c-Jun, activator protein 1 (AP-1) subunits, which turn on the transcription of the HO-1 gene by regulating the HO-1 promoter. 

ROS can also be involved in the therapeutic action of some antitumoral drugs. 

Soltan et al. [6] evaluated the antitumoral action of a derivative of the plant extract indirubin, DKP-071, on cutaneous T-cell lymphoma (CTCL). DKP-071 activated the extrinsic apoptosis cascade via caspase-8 and caspase-3 through downregulation of the caspase antagonistic proteins c-FLIP and XIAP. In response to DPK-071 treatment, a strong increase of ROS levels was observed as an early effect. ROS turned out upstream of all other proapoptotic effects monitored. Thus, ROS appear as a highly active proapoptotic pathway in CTCL.

The antioxidant capacity to protect against oxidative stress-linked disease has been evaluated by Zhao et al. [7], who studied the protective effects of the treatment with artemisinin, an anti-malarial Chinese medicine, on SH-SY5Y and hippocampal neuronal cells treated with hydrogen peroxide (H_2_O_2_). Artemisinin prevents cell death at clinically relevant doses in a concentration-dependent manner. Artemisinin restored the nuclear morphology, prevented the increased intracellular ROS, and attenuated apoptosis. These data suggested that artemisinin protected neuronal cells. Similar results were obtained in primary cultured hippocampal neurons. Cumulatively, these results indicated that artemisinin protected neuronal cells from oxidative damage, at least in part through the activation of AMPK. These findings support the role of artemisinin as a potential therapeutic agent for neurodegenerative diseases.

Moreover, some reviews are presented in this Special Issue. 

Lévy et al. [8] reviewed the current literature concerning the link between oxidative stress and protein aggregation processes, which are involved in the development of proteinopathies, such as Alzheimer’s disease, Parkinson’s disease, and prion disease. 

Damiano et al. [9] examined the data concerning antioxidant supplementation associated with exercise in normal and sarcopenic subjects. In older people, malnutrition and physical inactivity can lead to sarcopenia, a process in which oxidative stress seems to be involved. The effects of exercise and antioxidant dietary supplements in limiting age-related muscle mass loss and performance reduction have been evaluated in many studies but the results are conflicting. This can be due to the dual effects of ROS in skeletal muscle, which at low levels increase muscle force and induce adaptations to exercise, but at higher levels lead to a muscle performance decline. Therefore, the controversial results obtained with antioxidant supplementation in older persons could, in part, reflect the lack of univocal effects of ROS on muscle mass and function. 

Xiao and Meierhofer [10] reviewed the current knowledge about the three main renal cell carcinoma (RCC) subtypes—clear cell RCC (ccRCC), papillary RCC (pRCC), and chromophobe RCC (chRCC)—and highlight their mutual influence on GSH metabolism. Altered GSH metabolism contributes to the development and progression of the three renal carcinomas. All RCCs have a reduced oxidative phosphorylation capacity, and the respiratory chain is the main source of ROS. Raised oxidative stress levels in RCCs are counteracted by increased GSH levels that foster the survival of the malignancy. New studies have shown that combinatory therapy targeting two independent pathways of GSH synthesis and one involved in ROS metabolism is the key to improving the survival rate and eventually curing RCC.

Siauciunaite et al. [11] summarized the actual knowledge about the role of ROS as signaling molecules and key regulators of gene expression from an evolutionary point of view. They described recent work that has revealed significant species-specific differences in the gene expression response to ROS by exploring diverse organisms. This evidence supports the notion that during evolution, rather than being highly conserved, there is inherent plasticity in the molecular mechanisms responding to oxidative stress.

The review of Di Meo et al. [12] analyzed the literature dealing with sources of ROS production and the most important redox signaling pathways, including MAPKs that are involved in the responses to acute and chronic exercise in the muscle, particularly those involved in the induction of antioxidant enzymes.

Ismail et al. [13] collected and discussed studies analyzing the involvement of mitochondrial peroxiredoxins (Prdxs) in human cancers. They focused on signaling involving ROS and mitochondrial Prdxs that is associated with cancer development and progression. An upregulated expression of Prdx3 and Prdx5 has been reported in different cancer types, such as breast, ovarian, endometrial, and lung cancers, as well as in Hodgkin’s lymphoma and hepatocellular carcinoma. It is depicted that mitochondrial Prdxs are upregulated in a variety of cancer types and directly or indirectly regulated by transcription factors, microRNAs, and oncogenes. 

It is our opinion that the articles included in this Special Issue, despite dealing with such different topics, represent an important contribution to the knowledge of the physiological and pathological role of ROS, and give some information on the benefits and limitations of antioxidant treatment. 
